# Distinct roles of basal forebrain cholinergic neurons in spatial and object recognition memory

**DOI:** 10.1038/srep13158

**Published:** 2015-08-06

**Authors:** Kana Okada, Kayo Nishizawa, Tomoko Kobayashi, Shogo Sakata, Kazuto Kobayashi

**Affiliations:** 1 Department of Behavioural Sciences, Graduate School of Integrated Arts & Sciences, Hiroshima University, Higashi-Hiroshima 739-8521, Japan; 2 Department of Molecular Genetics, Institute of Biomedical Sciences, Fukushima Medical University School of Medicine, Fukushima 960-1295, Japan; 3 Japan Science and Technology Agency, Core Research for Evolutional Science and Technology, Kawaguchi 332-0012, Japan

## Abstract

Recognition memory requires processing of various types of information such as objects and locations. Impairment in recognition memory is a prominent feature of amnesia and a symptom of Alzheimer’s disease (AD). Basal forebrain cholinergic neurons contain two major groups, one localized in the medial septum (MS)/vertical diagonal band of Broca (vDB), and the other in the nucleus basalis magnocellularis (NBM). The roles of these cell groups in recognition memory have been debated, and it remains unclear how they contribute to it. We use a genetic cell targeting technique to selectively eliminate cholinergic cell groups and then test spatial and object recognition memory through different behavioural tasks. Eliminating MS/vDB neurons impairs spatial but not object recognition memory in the reference and working memory tasks, whereas NBM elimination undermines only object recognition memory in the working memory task. These impairments are restored by treatment with acetylcholinesterase inhibitors, anti-dementia drugs for AD. Our results highlight that MS/vDB and NBM cholinergic neurons are not only implicated in recognition memory but also have essential roles in different types of recognition memory.

Recognition memory is the fundamental ability to recognize a previously experienced stimulus. It is based on the contextual recollection of the stimulus and relative familiarity with it^1^,^2^, which requires processing various types of information such as objects and locations^3^. Previous studies show that different recognition memory processes are mediated through distinct brain regions, especially the hippocampus and several cortical areas, including the perirhinal and medial prefrontal cortices^4^,^5^,^6^. Impaired recognition memory is a prominent feature of amnesia and a symptom of Alzheimer’s disease (AD). AD is accompanied by a substantial loss of neurons containing acetylcholine in the basal forebrain^7^,^8^, which suggests that these cholinergic systems play a key role in recognition memory.

Basal forebrain cholinergic systems constitute discrete cell groups that innervate numerous brain regions. Neurons in the medial septum (MS) and vertical diagonal band of Broca (vDB) predominantly extend their axons to the hippocampus, forming the septo-hippocampal pathways, whereas neurons in the nucleus basalis magnocellularis (NBM) provide the main innervations to the entire cortex, forming the baso-cortical pathways^9^. The roles of the MS/vDB and NBM cholinergic neurons in recognition memory have been debated^10^,^11^. Previous studies on brain lesions indicate the importance of basal forebrain neurons in spatial reference and working memory in various maze tasks^12^,^13^,^14^,^15^. The anti-neuronal toxin 192-IgG saporin, which contains a monoclonal antibody against rat p75 neurotrophin receptor conjugated to the ribosome-inactivating protein^16^, has been used for selective ablation of basal forebrain cholinergic neurons, but its effects on behaviour are controversial. Ablation of MS/vDB or NBM neurons by 192-IgG saporin in rats resulted in impaired spatial memory in the water or radial maze task^17^,^18^, but other studies have not reproduced the results^19^,^20^,^21^. The effects of MS/vDB ablation on spatial working memory are also inconsistent between studies^19^,^20^,^22^,^23^,^24^,^25^,^26^, although NBM ablation does not appear to alter mnemonic function^19^,^26^,^27^. Therefore, it remains uncertain how basal forebrain cholinergic cell groups are involved in recognition memory. 192-IgG saporin toxicity seems to be accompanied by the loss of noncholinergic neurons and extensive tissue damage in the basal forebrain under certain conditions^19^,^21^,^25^,^26^. The side effects of this neurotoxin may influence the behavioural consequences.

In the present study, we used immunotoxin (IT)-mediated cell targeting to address the role of basal forebrain cholinergic neurons in recognition memory. This targeting enables selective elimination of target cell types based on the specificity of a recombinant IT, anti-Tac(Fv)-PE38^28^,^29^. We then tested spatial and object recognition memory by using reference and working memory tasks. IT injection into the MS/vDB or NBM selectively removed the respective cholinergic system in transgenic (Tg) mice. Elimination of MS/vDB cholinergic neurons impaired spatial but not object recognition memory in both the reference and working memory tasks. The NBM cholinergic elimination undermined only object recognition memory in the working memory task. These memory impairments were restored by cholinergic activation with inhibitors for acetylcholine metabolism. Our results indicate that MS/vDB and NBM neurons possess important roles in distinct types of recognition memory.

## Results

### Selective targeting of MS/vDB and NBM cholinergic cell groups

**Figure 1:**
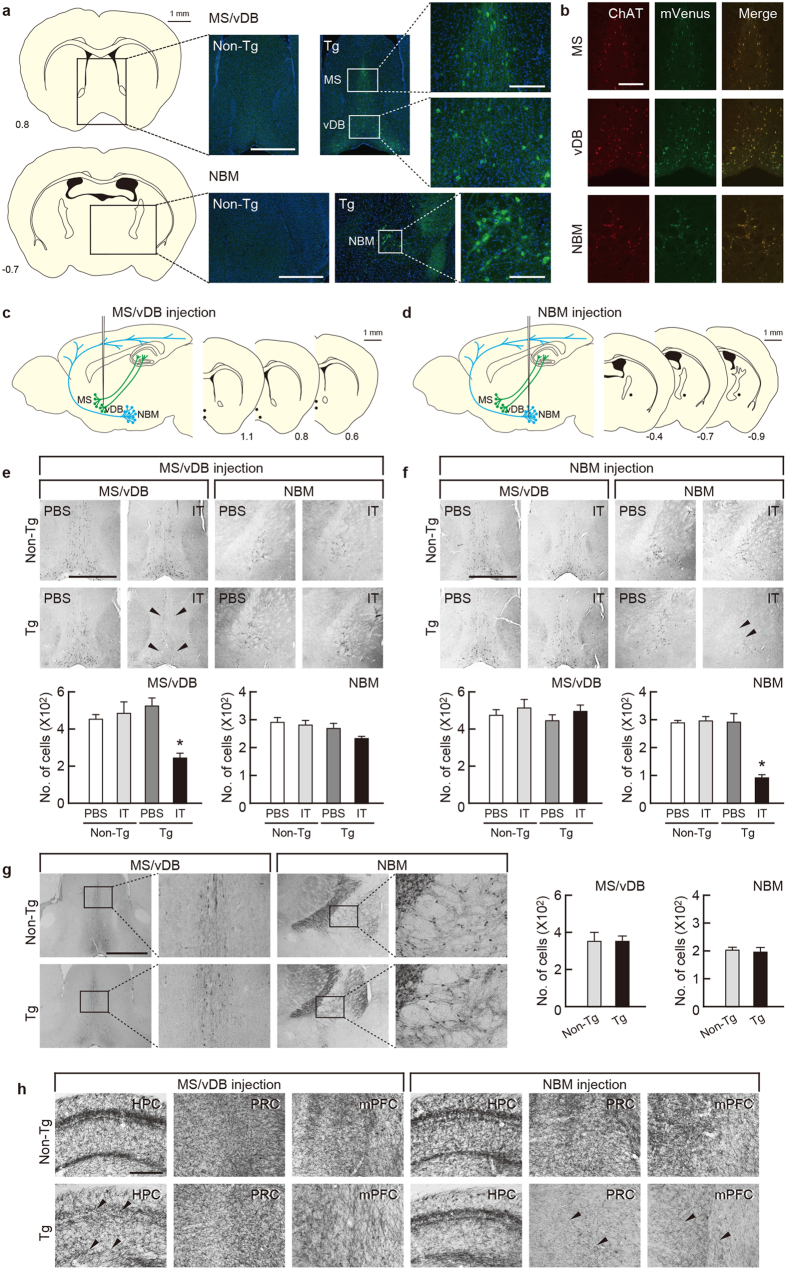
Selective targeting of cholinergic cell groups in the basal forebrain. (**a**) Expression of IL-2Rα/mVenus products in the basal forebrain in the Tg mice revealed by immunostaining for mVenus with sections from the MS/vDB and NBM. Scale bars: 200 μm. The brain areas corresponding to light microscopic images are shown in sections modified from an atlas of the mouse brain (Allen Mouse Brain Atlas. Available from: http://mouse.brain-map.org/)^53^. (**b**) Cell type-specific expression of transgene products in cholinergic neurons in the MS, vDB, and NMB shown by double immunofluorescence histochemistry for mVenus and ChAT. In a merged image, green signals (mVenus) overlap with red signals (ChAT), thus emitting yellow signals. Scale bars: 200 μm. **(c,d**) Intracranial injection by using stereotaxic surgery into the MS/vDB (**c**) or NBM (**d**). Two cholinergic systems are schematically illustrated in the left panel and injection coordinates are indicated in the right panel in sections modified from the Allen Mouse Brain Atlas http://mouse.brain-map.org/)^53^. The anteroposterior coordinates (mm) from bregma are shown. Scale bars: 1 mm. (**e,f**) Immunohistochemical staining for ChAT with sections prepared from the mice 7 days after IT or PBS injection into the MS/vDB (**e**) or NBM (**f**). Scale bars: 1 mm. Lower panels show cell counts of ChAT-positive neurons. Data are presented as mean ± s.e.m. *n *= 5 for each group. *P < 0.05 vs each of other three groups (Bonferroni method). (**g**) Immunostaining for parvalbumin with sections through the MS/vDB and NBM prepared from the IT-injected mice into the MS/vDB or NBM. Scale bars: 1 mm. Right panel shows cell counts of parvalbumin-positive cells. Data are presented as mean ± s.e.m. *n *= 5 for each group. (**h**) AChE staining with sections through the hippocampus (HPC), perirhinal cortex (PRC), and medial prefrontal cortex (mPFC) prepared from the IT-injected mice into the MS/vDB or NBM. Scale bars: 200 μm.

We performed the selective elimination of cholinergic neurons in the basal forebrain by using IT-mediated cell targeting^28^,^29^. The recombinant IT used was anti-Tac(Fv)-PE38, which consists of single-chain variable regions of a monoclonal antibody for human interleukin-2 receptor α-subunit (IL-2Rα) fused to a bacterial exotoxin catalytic fragment. The transgene construct contained the gene cassette encoding human IL-2Rα fused to a variant of enhanced yellow fluorescent protein (mVenus) introduced into the initiation codon site of the gene encoding choline acetyltransferase (ChAT) in a bacterial artificial chromosome clone^30^ (see Supplementary Fig. S1). The microinjection technique was used to generate multiple independent Tg mouse founders, and one strain, termed *ChAT-IL-2Rα/mVenus997-6*, expressed the IL-2Rα/mVenus transgene in both the Ms/vDB and NBM regions (Fig. 1a). Double immunofluorescence histochemistry detected mVenus-positive signals in the majority of ChAT-positive neurons, and overlapping signal ratios were 93% and 98% in the MS/vDB and NBM, respectively (Fig. 1b).

The Tg and non-Tg mouse littermates were given intracranial injections of IT (20 μg/ml) or phosphate-buffered saline (PBS) into the MS/vDB (0.2 μl × 12 sites) or NBM (0.3 μl × 6 sites) (Fig. 1c,d). One week after the surgery, brains were processed for immunohistochemistry. The basal forebrain sections were stained by using anti-ChAT antibody and viewed for cell counts. The IT injection into the MS/vDB or NBM resulted in a loss of ChAT-positive neurons in the corresponding regions in the Tg mice (Fig. 1e,f). One-way analysis of variance (ANOVA) for the mice that received injection into the MS/vDB indicated a significant difference in the number of MS/vDB cells among the four mouse groups (Fig. 1e; *F*_3,16 _= 9.509, *P *< 0.001), with a significant reduction in the IT-injected Tg mice compared with each of the other three groups (*P *< 0.05, Bonferroni method). However, no significant difference in the number of NBM cells occurred among the mouse groups (Fig. 1e). For the mice injected into the NBM, one-way ANOVA followed by *post hoc* multiple comparisons revealed that the NBM cell numbers were significantly lower in the IT-injected Tg mice compared with each of other three groups (Fig. 1f; *F*_3,16 _= 32.960, *P *< 0.05, Bonferroni method), whereas there was no difference in the MS/vDB cell numbers among the groups (Fig. 1f).

Immunostaining for parvalbumin on sections from the IT-injected mice displayed intact GABAergic interneurons in the target areas in the Tg mice (Fig. 1g). Cresyl violet staining of the sections showed no grossly visible damage in the MS/vDB and NBM in the Tg mice (Supplementary Fig. S2). Brain tissue injury was not observed even at a later stage, 6 months after the IT injection (Supplementary Fig. S2). To confirm the elimination of the septo-hippocampal and baso-cortical cholinergic pathways, we carried out acetylcholinesterase (AChE) staining with sections from the IT-injected mice. With IT injection into the MS/vDB, synaptic terminals with AChE-positive signals were markedly decreased in the hippocampus but not in the perirhinal and medial prefrontal cortices in the Tg mice (Fig. 1h). For the NBM injection, the positive terminals were decreased in the cortical areas but not in the hippocampus in the Tg mice (Fig. 1h). These data indicate the selective, efficient elimination of cholinergic neurons in the basal forebrain regions of Tg mice after IT injection.

### MS/vDB cholinergic elimination results in impaired spatial reference memory

**Figure 2:**
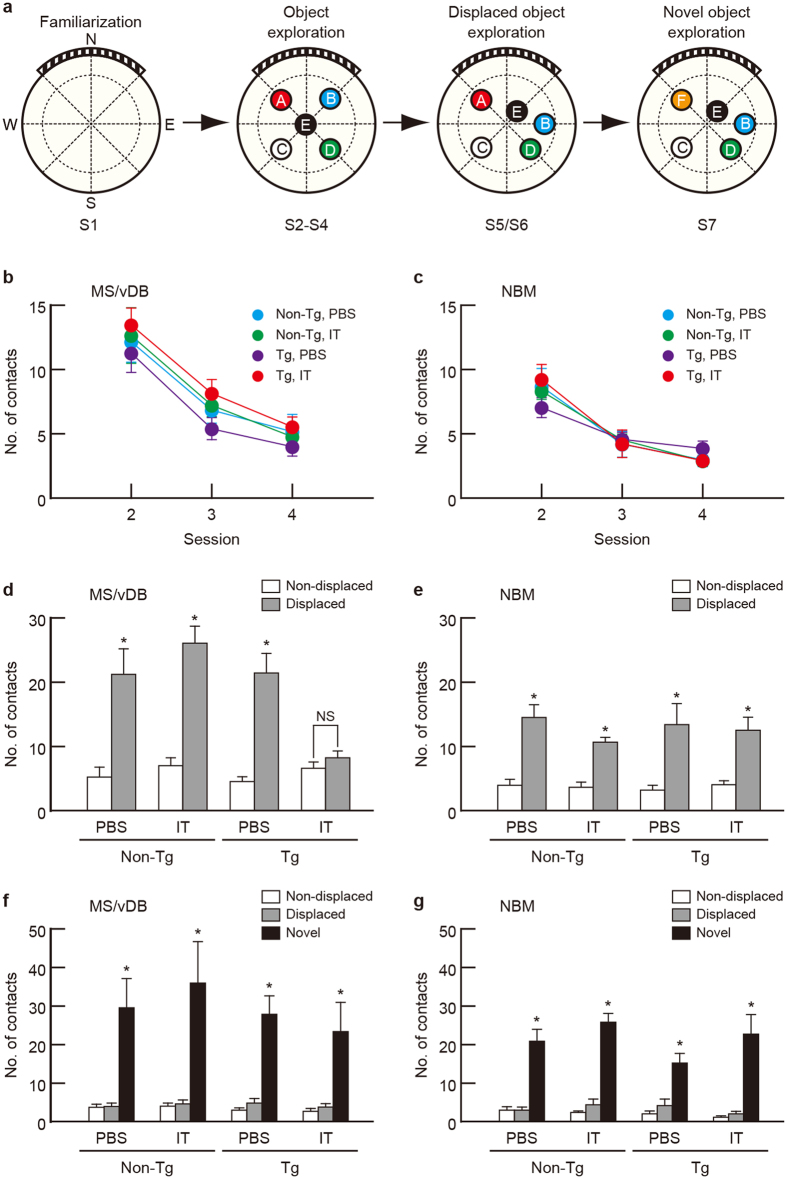
Targeting of the MS/vDB cholinergic neurons results in the impaired spatial reference memory. (**a**) Strategy for the serial object exploration task. A large circle indicates the circular open field with a striped board on the wall. Broken lines divide the open field into unit areas to measure locomotor activity. Mice individually explore the open field during seven successive sessions (S1–S7) that include the familiarization (S1), object exploration (S2–S4), displaced object exploration (S5/S6), and novel object exploration (S7) phases. Five different objects (**A–E**) were placed in the open field for S2–S4. Two objects (**B,E**) among these were displaced for S5/S6, and then one object (**A**) was exchanged by a novel object (**F**) in S7. (**b,c**) Mean number of contacts with objects during S2–S4. Tg and non-Tg mice were injected with IT solution or PBS into the MS/vDB (**b**) or NBM (**c**) and used for the task. Data are presented as mean ± s.e.m. *n *= 8 for each group. (**d,e**) Mean number of contacts with the non-displaced and displaced objects on a per-object basis in the spatial recognition test (S5). Mice injected with IT solution or PBS into the MS/vDB (**d**) or NBM (**e**) were used. Data are presented as mean ± s.e.m. *n *= 8 for each group. *P < 0.05 vs non-displaced object. NS, not significant. (**f,g**) Mean number of contacts with the non-displaced, displaced, and novel objects on a per-object basis in the object recognition test (S7). Mice injected with IT solution or PBS into the MS/vDB (**f**) or NBM (**g**) were used. Data are presented as mean ± s.e.m. *n *= 8 for each group. *P < 0.05 vs either non-displaced or displaced object.

To evaluate the impact of selective elimination of basal forebrain cholinergic neurons on spatial and object recognition memory in the reference memory task, we conducted a serial object exploration task^31^,^32^. The task included seven successive sessions (S1–S7) consisting of the following four phases: (1) familiarization (S1), (2) object exploration (S2–S4), (3) displaced object exploration (S5/S6), and (4) novel object exploration (S7) (Fig. 2a). Mice were first familiarized in the open field during S1, and then explored the field, which contained five different objects, during S2–S4. Among these objects, two were displaced for S5/S6 and one was replaced with a novel object for S7. The number of contacts with objects was counted during each session except for S1. The number of contacts during S5 and S7 was used to assess spatial and object reference memory, respectively.

The Tg and non-Tg littermates received a bilateral injection with IT solution or PBS into the MS/vDB or NBM and underwent the serial object exploration task. Measurement of unit crossings in the open field during each session showed normal locomotor activity in the IT-injected Tg mice (see Supplementary Fig. S3). Throughout the sessions (S2–S4), the number of contacts with objects was similarly reduced among the four mouse groups that were given the injection into the MS/vDB (Fig. 2b; group, *F*_3,28 _= 0.886, *P *= 0.460; session, *F*_2,56 _= 66.635, *P *< 0.001; interaction, *F*_6,56 _= 0.113, *P *= 0.993; two-way repeated-measures ANOVA) or NBM (Fig. 2c; group, *F*_3,28 _= 0.034, *P *= 0.991; session, *F*_2,56 _= 85.864, *P *< 0.001; interaction, *F*_6,56 _= 1.582, *P *= 0.169; two-way repeated measures ANOVA), showing similar habituation to the objects among the mouse groups injected into the two basal forebrain regions.

In the spatial reference memory test (S5), two-way ANOVA of the number of contacts in mice that received the MS/vDB injection indicated a significant main effect of group (*F*_3,28 _= 4.334, *P *= 0.012) and object (*F*_1,28 _= 126.688, *P *< 0.001) with a significant group × object interaction (*F*_3,28 _= 11.150, *P *< 0.001) (Fig. 2d). The number of contacts with the displaced objects was significantly higher than with the non-displaced objects for the PBS- or IT-injected non-Tg and PBS-injected Tg mice (*P *< 0.05, Bonferroni method), whereas the contacts with the displaced and non-displaced objects did not differ in the IT-injected Tg mice. For the mice with the NBM injection, the number of contacts with the displaced objects was higher than with the non-displaced objects in each mouse group (Fig. 2e; group, *F*_3,28 _= 0.446, *P *= 0.722; object, *F*_1,28 _= 88.718, *P *< 0.001; interaction, *F*_3,28 _= 0.722, *P *= 0.547; two-way ANOVA). Therefore, the deficit in spatial reference memory was found in the Tg mice with IT injection into the MS/vDB but not the NBM.

In the object reference memory test (S7), the number of contacts for the mice that were given the MS/vDB injection indicated a significant difference among objects (Fig. 2f; group, *F*_3,28 _= 0.545, *P *= 0.656; objects, *F*_2,56 _= 41.928, *P *< 0.001, interaction, *F*_6,56 _= 0.386, *P *= 0.885; two-way ANOVA), and the contacts with the novel object were higher compared to those with the familiar non-displaced or displaced object in each mouse group. For the mice with the NBM injection, the number of contacts was also significantly greater with the novel object than that with the other two objects in each group (Fig. 2g; group, *F*_3,28 _= 1.168, *P *= 0.340; object, *F*_2,56 _= 131.041, *P *< 0.001; interaction, *F*_6,56 _= 2.111, *P *= 0.066; two-way ANOVA). Thus, object reference memory was not altered in the IT-injected Tg mice into either the MS/vDB or the NBM. These results demonstrate that selective elimination of cholinergic neurons in the MS/vDB but not the NBM results in impaired spatial recognition memory in the reference memory task, while elimination of the MS/vDB or NBM neurons does not influence object recognition memory in the same task.

In the serial object exploration task experiment, we measured the time spent in contacts with the objects in addition to the number of contacts during the sessions (see Supplementary Fig. S4). The analysis of the time spent in contacts with the objects yielded conclusions similar to those for the number of contacts. Spatial reference memory was disrupted in the Tg mice that received an IT injection into the MS/vDB but not those that received the injection into the NBM (compare Fig. 2d,e to Supplementary Figs S4c and S4d, respectively); however, object reference memory was not changed in Tg animals receiving the IT injection into either of the two cholinergic neuron areas (compare Fig. 2f,g to Supplementary Figs S4e and S4f, respectively). Therefore, we used the number of contacts with objects to evaluate recognition memory in the mouse groups in the following experiments.

### MS/vDB and NBM cholinergic ablation differentially disturbs working memory

**Figure 3:**
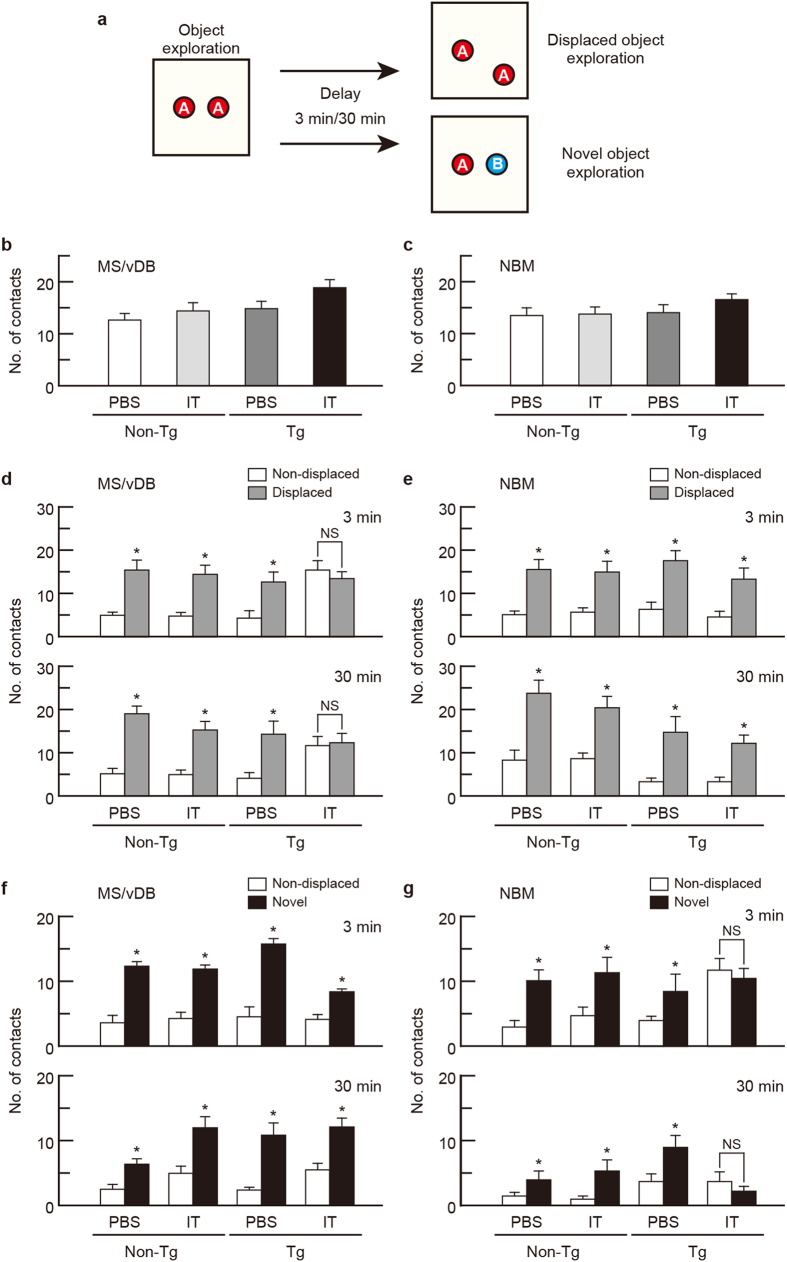
Elimination of the MS/vDB and NBM cholinergic neurons impaired spatial and object working memory, respectively. (**a**) Strategy for the one-trial object exploration task. A large square represents the square open field. Mice individually explored the open field during the object exploration and displaced/novel object exploration sessions with an intersession interval of 3 or 30 min. Two identical objects (**A**) were placed for the exploration session, and one object was displaced or exchanged by a novel object (**B**). (**b,c**) Mean number of contacts during the object exploration session. Tg and non-Tg mice were injected with IT solution or PBS into the MS/vDB (**b**) or NBM (**c**) and used for the task. Data are presented as mean ± s.e.m. *n *= 8 for each group. (**d,e**) Mean number of contacts with the non-displaced and displaced objects in the spatial recognition test (displaced object exploration session). Mice injected with IT solution or PBS into the MS/vDB (**d**) or NBM (**e**) were used. Data are presented as mean ± s.e.m. *n *= 8 for each group. *P < 0.05 vs non-displaced object (Bonferroni method). NS, not significant. (**f,g**) Mean number of contacts with the non-displaced and novel objects in the object recognition test (novel object exploration session). Mice injected with IT solution or PBS into the MS/vDB (**f**) or NBM (**g**) were used. Data are presented as mean ± s.e.m. *n *= 8 for each group. *P < 0.05 vs non-displaced object. NS, not significant.

To validate the influence of basal forebrain cholinergic elimination on spatial and object recognition memory in the working memory task, we used a one-trial object exploration task^33^,^34^, which consisted of two sessions: (1) object exploration and (2) displaced/novel object exploration (Fig. 3a). Mice first explored the open field in which two identical objects were placed. At different intersession delay periods (3 and 30 min), the mice again explored the field, in which one object had been displaced or replaced by a novel object. The number of contacts with objects was counted during each session, and this number was used to validate spatial and object working memory from the displaced and novel object exploration sessions, respectively.

The Tg and non-Tg mice were given a bilateral injection with IT solution or PBS into the MS/vDB or NBM, and then subjected to the one-trial object exploration task. The number of contacts with objects during the exploration session was indistinguishable among the four mouse groups injected into the MS/vDB (Fig. 3b; *F*_3,28 _= 2.298, *P *= 0.099, one-way ANOVA) or NBM (Fig. 3c; *F*_3,28 _= 0.542, *P *= 0.658, one-way ANOVA). In the spatial working memory test, two-way ANOVA of the number of contacts for the mice with the MS/vDB injection indicated a significant main effect of group (*F*_3,28 _= 4.009, *P *= 0.017 for 3 min, *F*_3,28 _= 0.753, *P *= 0.530 for 30 min) and object (*F*_1,28 _= 33.166, *P *< 0.001 for 3 min, *F*_1,28 _= 109.646, *P *< 0.001 for 30 min) with a significant interaction (*F*_3,28 _= 6.389, *P *= 0.002 for 3 min, *F*_3,28 _= 11.514, *P *< 0.001 for 30 min) (Fig. 3d). The contact number for the displaced object was significantly higher than for the non-displaced object in the PBS- or IT-injected non-Tg and PBS-injected Tg mice in each delay period (*P *< 0.05), whereas the number for the displaced and non-displaced objects was unaffected in the IT-injected Tg mice. For the mice injected into the NBM, the contact number for the displaced objects was elevated relative to that for the non-displaced objects in each mouse group (Fig. 3e; group, *F*_3,28 _= 0.573, *P *= 0.637; object, *F*_1,28 _= 108.635, *P *< 0.001; interaction, *F*_3,28 _= 0.360, *P *= 0.783 for 3 min; group, *F*_3,28 _= 4.795, *P *= 0.008; object, *F*_1,28 _= 102.567, *P *< 0.001; interaction, *F*_3,28 _= 1.344, *P *= 0.280 for 30 min; two-way ANOVA). Therefore, spatial working memory was impaired only in the IT-injected Tg mice into the MS/vDB but not into the NBM.

In the object working memory test, for mice that received the injection into the MS/vDB, the number of contacts with the novel object was greater than with the familiar, non-displaced object in the four mouse groups at both delay periods (Fig. 3f; *F*_3,28 _= 1.076, *P *= 0.375; object, *F*_1,28 _= 69.524, *P *< 0.001; interaction, *F*_3,28 _= 2.311, *P *= 0.098 for 3 min; group, *F*_3,28 _= 2.893, *P *= 0.053; object, *F*_1,28 _= 69.238, *P *< 0.001; interaction, *F*_3,28 _= 1.524, *P *= 0.230 for 30 min; two-way ANOVA). For the NBM injection, the contact number for the novel object was significantly higher than for the non-displaced object in the PBS- or IT-injected non-Tg and PBS-injected Tg mice in each delay period (Fig. 3g; group, *F*_3,28 _= 2.182, *P *= 0.112, object, *F*_1,28 _= 28.085, *P *< 0.001, interaction, *F*_3,28 _= 5.730, *P *= 0.003 for 3 min; group, *F*_3,28 _= 2.518, *P *= 0.078, object, *F*_1,28 _= 19.078, *P *< 0.001, interaction, *F*_3,28 _= 6.079, *P *= 0.003 for 30 min; two-way ANOVA; *P *< 0.05), but the number for the novel and non-displaced objects exhibited no change in the IT-injected Tg mice. Thus, object working memory was affected in the IT-injected Tg mice into the NBM but not into the MS/vDB. The data highlight that selective targeting of cholinergic neurons in the MS/vDB and NBM differentially disrupts spatial and object recognition memory in the working memory task.

### Recovery of recognition memory deficits by AChE inhibition

To confirm the involvement of basal forebrain cholinergic neurons in recognition memory, we examined whether the memory impairment in mice lacking cholinergic cell groups in the basal forebrain could be recovered by treatment with AChE inhibitors donepezil (Done) and rivastigmine (Riva). Done and Riva are anti-dementia drugs for AD^35^ and are also effective on the memory deficits in AD models of rodents^36^.

**Figure 4:**
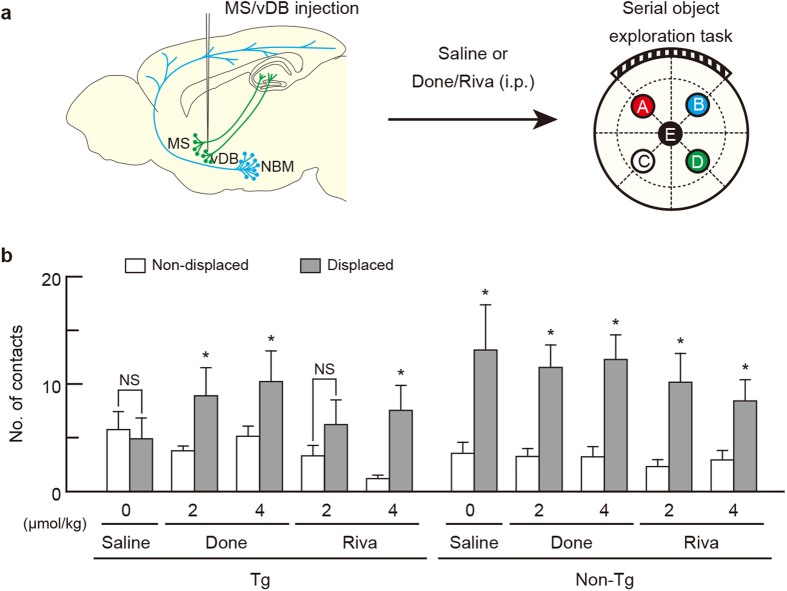
Drug recovery of spatial reference memory deficit in mice lacking MS/vDB cholinergic neurons. (**a**) Experimental design for recovery of memory deficit. Mice were injected with IT solution into the MS/vDB, administered i.p. with saline or Done/Riva (2 and 4 μmol/kg), and tested for spatial reference memory with the serial object exploration task. Two cholinergic systems are schematically illustrated in in sections modified from the Allen Mouse Brain Atlas (http://mouse.brain-map.org/)^53^. (**b**) Mean number of contacts with the non-displaced and displaced objects on a per-object basis in the spatial recognition test. Data are presented as mean ± s.e.m. *n *= 8 for each group. *P < 0.05 vs non-displaced object (Bonferroni method). NS, not significant.

First, we tested the recovery of the deficit in spatial reference memory in the mice lacking the MS/vDB cholinergic neurons. The Tg and non-Tg mice were given the IT solution bilaterally into the MS/vDB, administered saline or different doses of Done and Riva (2 and 4 μmol/kg) intraperitoneally (i.p.), and then used for the serial object exploration task (Fig. 4a). In the spatial reference memory test, the number of contacts with the displaced objects was significantly increased compared to that with the non-displaced objects in the Tg mice that received high/low doses of Done and high dose of Riva (Fig. 4b; group: *F*_9,70 _= 0.720, *P *= 0.689; objects: *F*_1,70 _= 78.917, *P *< 0.001; interaction: *F*_9,70 _= 2.256, *P *= 0.028; two-way ANOVA; *P *< 0.05), although the contact number for the two objects was not altered in the Tg mice that were administered saline. In the non-Tg mice, the number for the displaced objects was significantly higher than for the non-displaced objects in all mouse groups (*P *< 0.05). These data show that the deficit in spatial reference memory in mice lacking the MS/vDB cholinergic neurons can be recovered by the treatment with AChE inhibitors.

**Figure 5:**
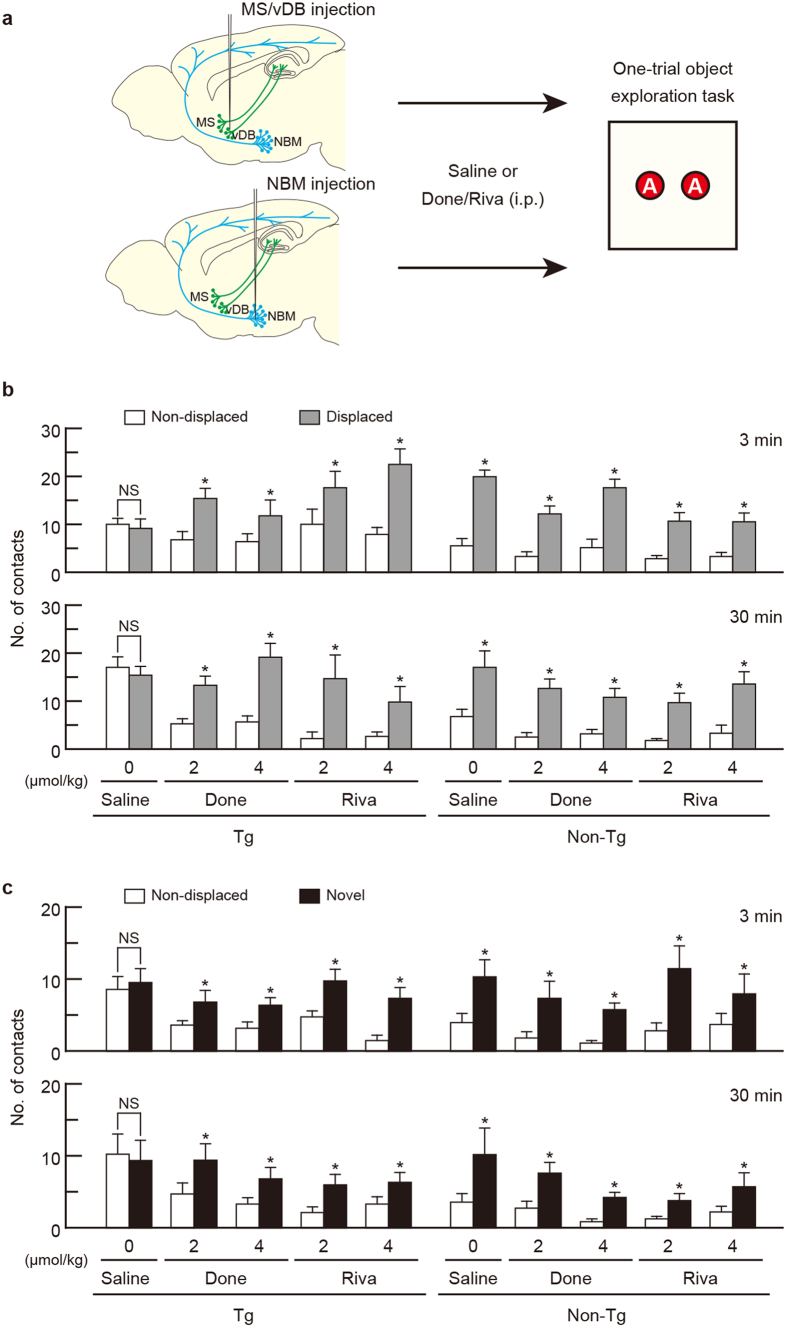
Drug restoration of spatial and object working memory defects in mice lacking MS/vDB or NBM cholinergic neurons. (**a**) Experimental design for restoration of memory defects. Mice were injected with IT solution into the MS/vDB or NBM, administered i.p. with saline or Done/Riva (2 and 4 μmol/kg), and tested for spatial and object working memory with the one-trial object exploration task. The cholinergic systems are schematically illustrated in in sections modified from the Allen Mouse Brain Atlas (http://mouse.brain-map.org/)^53^. (**b**) Mean number of contacts with the non-displaced and displaced objects on a per-object basis in the spatial recognition test with 3- or 30-min delay period. The mice injected with IT solution into the MS/vDB were used. Data are presented as mean ± s.e.m. *n *= 8 for each group. *P < 0.05 vs non-displaced object (Bonferroni method). NS, not significant. (**c**) Mean number of contacts with the non-displaced and novel objects on a per-object basis in the object recognition test with 3- or 30-min delay period. The mice injected with IT solution into the NBM were used. Data are presented as mean ± s.e.m. *n *= 8–12 for each group. *P < 0.05 vs non-displaced object (Bonferroni method). NS, not significant.

Next, the mice were given IT solution bilaterally into the MS/vDB or NBM, treated with different doses of Done and Riva (2 and 4 μmol/kg i.p.), and then tested in the one-trial object exploration task (Fig. 5a). The mice with the injections into the MS/vDB and NBM were used for the tests of spatial and object working memory, respectively. In the spatial working memory test, the number of contacts with the displaced objects was higher compared to that with the non-displaced objects in the Tg mice that received high/low doses of Done and Riva (Fig. 5b; group: *F*_9,70 _= 4.274, *P *< 0.001; objects: *F*_1,70 _= 102.318, *P *< 0.001; interaction: *F*_9,70 _= 2.879, *P *= 0.006 for 3 min; group: *F*_9,70 _= 3.361, *P *= 0.002; objects: *F*_1,70 _= 134.641, *P *< 0.001; interaction: *F*_9,70 _= 3.174, *P *= 0.003 for 30 min; two-way ANOVA, *P *< 0.05), although the contacts with the two objects did not differ in the saline-treated Tg mice. In the non-Tg mice, the number for the displaced objects was significantly larger than for the non-displaced objects in all mouse groups (*P *< 0.05). In the object working memory test, the contacts with the novel objects showed an increment compared to those with the non-displaced objects in the Tg mice that received high/low doses of Done and Riva (Fig. 5c; group: *F*_9,88 _= 1.701, *P *= 0.101; objects: *F*_1,88 _= 105.810, *P *< 0.001; interaction: *F*_9,88 _= 2.104, *P *= 0.037 for 3 min; group: *F*_9,88 _= 2.267, *P *= 0.025; objects: *F*_1,88 _= 88.605, *P *< 0.001; interaction: *F*_9,88 _= 2.681, *P *= 0.008 for 30 min; two-way ANOVA, *P *< 0.05), though the contacts with the two objects were indistinguishable in the saline-treated Tg mice. In the non-Tg mice, the number for the novel objects was higher than for the non-displaced objects in all groups (*P *< 0.05). These data demonstrate the restoration of spatial and object working memory defects in mice lacking the MS/vDB and NBM cholinergic neurons through treatment with AChE inhibitors.

## Discussion

In the present study, we performed selective, efficient targeting of two major groups of basal forebrain cholinergic neurons by using IT-mediated cell targeting. We then tested spatial and object recognition memory in reference and working memory tasks. Selective elimination of the MS/vDB cholinergic neurons resulted in impaired spatial recognition memory in both the reference and working memory tasks but not object recognition memory in these tasks. The NBM cholinergic elimination impaired only object recognition memory in the working memory task, and showed no changes in other types of recognition memory. In addition, these memory impairments were recovered by cholinergic activation through treatment with AChE inhibitors. Our results demonstrate that the MS/vDB and NBM cholinergic cell groups have critical roles in different types of recognition memory.

192-IgG saporin is reported to induce nonspecific damage of parvalbumin-positive GABAergic neurons in the basal forebrain and extensive tissue injury at later stages with higher doses (0.4–1 μg) for the intracranial injection^19^,^21^,^25^,^26^; lower doses (~0.2 μg for the intracranial or intracerebroventricular injection) appear to exclude these effects^37^,^38^. Another saporin-conjugated neurotoxin with a monoclonal antibody to murine p75 receptor has been produced for selective ablation of basal forebrain cholinergic neurons in mice^39^,^40^. However, this neurotoxin seems to be less potent than the 192-IgG saporin reacting to rat neurons^39^. In this study, we developed the Tg approach to selectively eliminate basal forebrain cholinergic neurons using a specific anti-Tac(Fv)-based recombinant IT. Intracranial injection of the IT (48 ng for the MS/vDB and 36 ng for the NBM, bilaterally) in Tg mice did not affect parvalbumin-positive GABAergic neurons in the basal forebrain, and no tissue damage occurred around the injection sites at either the early or late stages. Our approach used lower doses of injected IT than those used for saporin-conjugated neurotoxins, because the recombinant IT had a higher affinity for IL-2Rα and a smaller molecular weight (63 kDa) based on the single-chain antibody variable regions fused to a bacterial exotoxin catalytic domain. Our strategy for selective, efficient elimination of basal forebrain cholinergic neurons will lead to a clearer understanding of the physiological and behavioural functions of these cholinergic neurons.

Some previous studies of cholinergic ablation with 192-IgG saporin implicated the MS/vDB cell groups in spatial reference memory in the water or radial maze task^17^,^18^, but other studies did not support these results^19^,^20^,^21^. For spatial working memory, inconsistent reports show positive^19^,^20^,^22^,^25^,^26^ and negative^23^,^24^ data for the role of these cell groups. Our IT-mediated targeting of the MS/vDB cholinergic neurons impaired spatial recognition memory in both the reference and working memory tasks. These data clearly support the important roles of MS/vDB cholinergic neurons in the spatial recognition-based reference and working memory, suggesting that these neurons participate in memory processes related to spatial information despite the difference in the memory strength or frequency of stimulus presentation. The MS/vDB cholinergic neurons mainly innervate the hippocampal structure^9^, which is generally known to play a central role in spatial memory formation^41^. Acetylcholine release is increased in the hippocampus during exploration of a novel environment^42^. This evidence collectively suggests that the MS/vDB cholinergic neurons, mainly through the septo-hippocampal pathways, mediate the processing of reference and working memory related to spatial information.

Previous studies indicate the involvement of the NBM cholinergic neurons in spatial reference memory in the maze tasks^17^,^18^, but other studies report inconsistent results^19^,^21^. In addition, the NBM neurons do not appear to process mnemonic function^19^,^26^,^27^ and are likely to subserve attentional processes^43^,^44^. IT targeting of the NBM cholinergic neurons undermined only object recognition memory in the working memory task. The data support that the NBM cholinergic neurons do not process the spatial recognition-based reference and working memory; however, NBM neurons are required for the object recognition-based working memory, suggesting that the NBM contributes to short-term memory processing associated with familiarization with the objects. Since the NBM cholinergic neurons project to the entire cortex^9^, some cortical regions including the perirhinal and medial prefrontal cortices may be associated with the object working memory processes. The perirhinal cortex of the temporal lobe is crucial for object recognition memory after a certain delay period^45^,^46^. Although the removal of cholinergic inputs into the perirhinal cortex disrupts object recognition memory^47^, the origin of cholinergic neurons in the basal forebrain has not been identified. Pharmacological blockade of cholinergic transmission in the perirhinal cortex modulates object recognition memory, although the effects are stimulatory or inhibitory depending on the conditions^48^,^49^,^50^. These results suggest that the cholinergic system derived from the NBM through the baso-cortical pathways modulates the perirhinal cortical activity and then contributes to the formation of object working memory. The medial prefrontal cortex mediates integrated processes of object recognition memory through interactions with the perirhinal cortex and hippocampus^3^. In particular, a lesion of the medial prefrontal cortex disrupts “object-in-place” and “temporal order” recognition memory, suggesting the importance of this brain region in judgments of the sequence or order of object presentations^51^. In this study, we did not test these memory processes with the mice lacking basal forebrain cholinergic neurons. The behavioural role of NBM neurons through the medial prefrontal cortex remains to be examined.

AD is accompanied by a substantial loss of cholinergic neurons in the basal forebrain^7^,^8^. In this study, we undertook a successful strategy for selective elimination of basal forebrain cholinergic neurons through IT-mediated cell targeting, which resulted in various types of recognition memory being impaired. These impairments were restored by treatment with AChE inhibitors that are used as anti-dementia drugs for AD^35^. Our strategy thus provides a new Tg animal model with recognition memory impairment based on the selective loss of basal forebrain cholinergic neurons. It was more effective with lower injected doses compared with the saporin-conjugated neurotoxin and showed high selectivity for the target neuronal populations. This model will be useful for a better understanding of the neural mechanisms underlying cognitive impairments and for the development of approaches for diagnostic and therapeutic treatments for dementia.

## Methods

Tg mouse production. The transgene construct contained the gene cassette encoding IL-2Rα/mVenus followed by the SV40 early-gene polyadenylation signal in the place of the initiation codon in exon 3 of the *ChAT* gene^30^. The construct was linearized by *Sfi*I digestion, purified by pulse field gel electrophoresis, and microinjected into fertilized C57BL/6J mouse eggs, which were then implanted into pseudopregnant females. The *ChAT-IL-2Rα/mVenus* Tg mice were identified by Southern blot hybridization or PCR with genomic DNA prepared from tail clips. Tg and non-Tg littermates were used for the following experiments. All animal experiments were approved and performed in accordance with the guidelines for the care and use of laboratory animals established by the Animal Experiments Committee of Fukushima Medical University and Hiroshima University.

Intracranial surgery. Mice (8 weeks old) were anesthetized with sodium pentobarbital (50 mg/kg, i.p.) and subjected to bilateral intracranial injection of IT solution [20 μg/ml anti-Tac(Fv)-PE38 in PBS containing 0.1% mouse serum albumin]. For targeting of cholinergic neurons in the MS/vDB and NBM, solution was injected into 12 sites (0.2 μl/site) and 6 sites (0.3 μl/site), respectively, through a glass micropipette that was stereotaxically introduced by using the coordinates from an atlas of the mouse brain^52^. The anteroposterior, mediolateral and dorsoventral coordinates (mm) from bregma and dura were (1.1, ±0.1, −3.7), (1.1, ±0.1, −4.1), (0.8, ±0.1, −3.8), (0.8, ±0.3, −4.7), (0.6, ±0.1, −3.7), and (0.6, ±0.1, −4.2) for injection into the MS/vDB; and (−0.4, ±1.6, −3.7), (−0.7, ±1.8, −3.8), and (−0.9, ±2.0, −3.8) for injection into the NBM. Injection was carried out at a constant flow rate of 0.1 μl/min with a microinfusion pump, and the micropipette was left *in situ* for 2 min after each infusion.

Drug treatment. Donepezil hydrochloride (Sequoia Research Products Ltd.) and rivastigmine hydrogen tartrate (provided by Novartis Pharma AG, Basel, Switzerland) were dissolved into saline at a concentration of 0.2 or 0.4 mM. Mice received the intraperitoneal treatment of drug solution (2 or 4 μmol/kg) 30 min before the behavioural testing.

Histology. Fixed brains were cut into sections, and the sections were incubated with primary antibodies for GFP (rabbit, 1:2,000, Life Technologies), ChAT (mouse, 1:1,000, Millipore), parvalbumin (rabbit, 1:1000, Sigma-Aldrich), and then with fluorescein isothiocyanate-conjugated or biotinylated secondary antibodies. The immunoreactive signals were visualized by using a Vectastain Elite ABC kit. For double immunofluorescence histochemistry, the sections were incubated with anti-GFP and anti-ChAT antibodies, and then with species-specific secondary antibodies conjugated to Alexa488 (Molecular Probes) and Cy3 (Jackson ImmunoResearch). 4,6-Diamidino-2-phenylindole (DAPI, 1:1,000, Molecular Probes) was used to label nuclei. For cell counts, the number of immunopositive cells in each area was counted in the representative four sections through the MS/VDB or NBM (the anteroposterior coordinates from bregma: 1.3, 0.9, 0.7, and 0.5 mm for the MS/VDB; and −0.3, −0.5, −0.8, and −1.0 mm for the NBM), and the total number of immunopositive cells was calculated. Cresyl violet staining was processed to check for nonspecific damage on the brain tissue around the injection sites. For AChE staining, brain sections were rinsed in 0.1 M maleic acid buffer (pH = 6.0) and incubated for 10 min in 0.1 M maleic acid buffer containing 340 μM acetylthiocholine iodide, 50 μM sodium citrate, 30 μM cupric sulphate, and 5 μM potassium ferricyanide. To inhibit non-acetylcholinesterases, 10 nM ethopropazine was added to the solution. After the incubation, sections were washed with 50 mM Tris-HCl buffer (pH = 7.6) and soaked in 50 mM Tris-HCl buffer containing 620 nM cobalt chloride. Sections were then incubated for 7 min in 50 mM Tris-HCl buffer containing 190 μM diaminobenzidine, 0.003% H_2_O_2_, and 0.1% nickel ammonium sulphate.

Behavioural analysis. Adult naïve male mice were housed in standard lab Plexiglas cages (225 × 338 × 140 mm, length × width × height, four mice per cage) on a 12-h light/12-h dark cycle. The experiments were conducted during the light period. After the surgery, mice were given a 1-week recovery period, followed by the serial object exploration task^32^ or one-trial object exploration task^33^,^34^. Different mice were used for the serial object exploration task, one-trial object recognition task, and the experiment with drug treatments. The open fields for these tasks were positioned in the centere of a room that had overhead lighting and contained various visual cues, including a computer, monitor, and shelves and posters on the wall. The animals’ behaviour was monitored using an overhead colour CCD camera (AVC-636SN; ITS, Co. Ltd.) connected to a digital video cassette recorder. During the tasks, the number of times the mouse snout made contact with an object (i.e., number of contacts) was manually counted. The counted data were confirmed by the video-recorded behaviour. The measurements of exploration were scored by an observer who was blinded to the animal groups and drug treatments.

For the serial object exploration task, a circular, polyvinylchloride open field (70-cm in diameter, 40-cm high) was used, and four positions in the open field were marked as north (N), south (S), east (E), and west (W) (see Fig. 2a). The wall was equipped with a striped board composed of 2.5-cm-wide vertical black and white lines in the N position. The base of the open field was divided into four quadrants (NW, NE, SW, and SE), and further subdivided into 16 equal-sized areas for the measurement of locomotor activity. All of the objects (A–F) used for the task had different visual and haptic features. The task contained seven successive sessions (S1–S7; 6 min for each session) with an intersession interval of 3 min. These sessions consisted of four different phases for familiarization (S1), object exploration (S2–S4), displaced object exploration (S5/S6), and novel object exploration (S7). During S1, a mouse was placed in the empty open field and familiarized with it. During S2–S4, five different objects (A–E) were used, and four objects (A–D) were placed in the middle of each quadrant, while another object (E) was positioned in the centre of the field. Two objects (B and E) were displaced during S5, and no objects were moved during S6. Another object (A) was replaced by a novel object (F) during S7. A duplicate in the case of a repeating object was used for each phase within a complete trial. The mouse was allowed to explore freely in the open field during S2–S7, and the number of contacts with the objects was counted. In each session, the mouse began its exploration from one of four release points (N, S, E, and W) in a pseudo-random manner. Mice were placed back in their home cages during the 3-min intersession interval. Locomotor activity was assessed by counting the number of unit crossings with the subdivided areas in the base. During S5, the average of number of contacts with non-displaced objects (A, C, and D) or displaced objects (B and D) was calculated. During S7, the average number of contacts with non-displaced objects (C and D) or displaced objects (B and E) and the number of contacts with the novel object (F) were determined. All objects and the open field were washed with 70% ethanol after each trial.

For the one-trial object exploration task, a square, polyvinylchloride open field (35 × 35 cm and 30 cm high) was used (see Fig. 3a). The task consisted of two sessions (3 min for each session) for the object exploration and displaced/novel object exploration with an intersession delay period of 3 or 30 min. The sequencing of 3-min and 30-min delays after the object exploration was counterbalanced among the mice. During the object exploration session, two identical objects were placed in the open field in a line-shaped spatial configuration, and during the displaced/novel object exploration session one object was displaced or exchanged with a novel one (B). A duplicate in the case of a repeating object was used for each phase within a complete trial. The mice were allowed to explore freely in the open field during the sessions, and the number of contacts with the objects was counted. In each session, the mice began their exploration from one of two release points in a pseudo-random manner and were placed back in their home cages during the 3- or 30-min intersession delay period. The left–right positions of the displaced and non-displaced/novel objects were counterbalanced. The open field and objects were washed in 70% ethanol after each trial.

Statistical analysis. For statistical comparisons, the ANOVA and *post hoc* Bonferroni test were used with significance set at *P *< 0.05. All values were expressed as the mean ± s.e.m. of the data. Repeated ANOVA was used for the analysis of within-subjects design.

## Additional Information

**How to cite this article**: Okada, K. *et al.* Distinct roles of basal forebrain cholinergic neurons in spatial and object recognition memory. *Sci. Rep.* **5**, 13158; doi: 10.1038/srep13158 (2015).

## Supplementary Material

Supplementary Information
