# Supplementary material for: Distinct roles of basal forebrain cholinergic neurons in spatial and object recognition memory
Source: Sci Rep. 2015 Aug 6;5:13158. doi: 10.1038/srep13158 (PMC4526880; doi:10.1038/srep13158)
Supplement: Supplementary Information [file srep13158-s1.pdf]

SUPPLEMENTARY INFORMATION

Title: Distinct roles of basal forebrain cholinergic neurons in spatial and object recognition memory

Authors: Kana Okada, Kayo Nishizawa, Tomoko Kobayashi, Shogo Sakata, and Kazuto Kobayashi

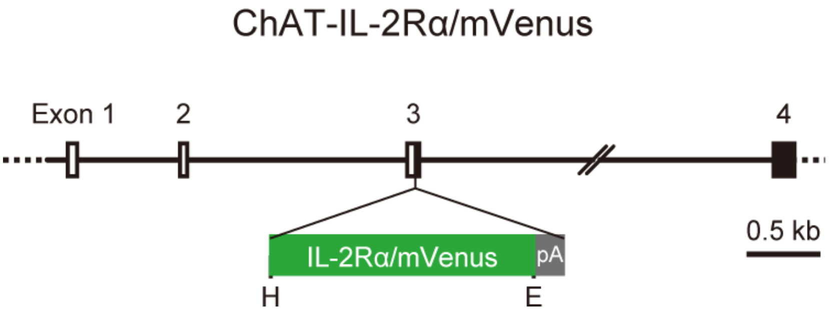

Supplementary Figure S1. The transgene construct used for transgenic rat production. The transgene construct contained the human IL-2RŬ/mVenus fusion protein under the control of the *ChAT* gene promoter. Exons 1ï 4 of the rat *ChAT* gene are shown. Open and closed boxes indicate the 5Njnoncoding region and coding regions, respectively. Restriction enzyme abbreviations: E, *Eco*RI; H, *Hin*dIII. pA; polyadenylation signal from the SV40 early-gene.

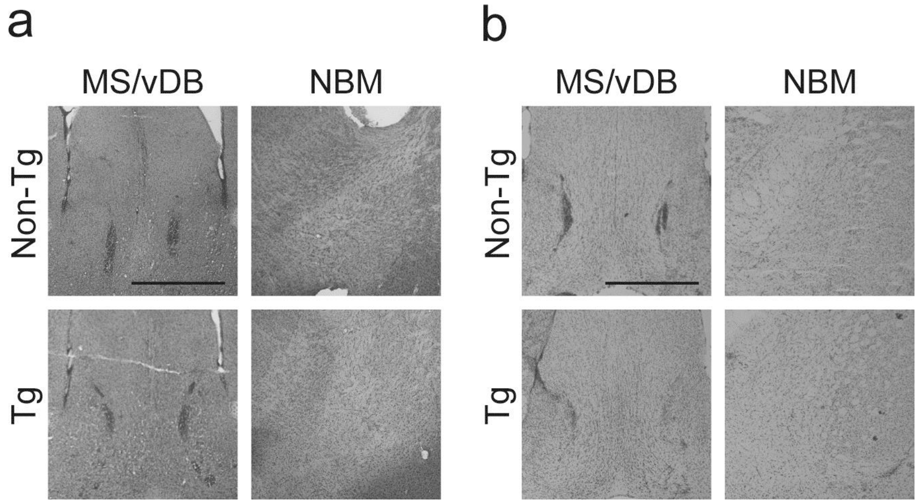

Supplementary Figure S2. Cresyl violet staining with brain sections. Sections from the MS/vDB or NBM were prepared from the non-Tg and Tg mice 7 days (**a**) or 6 months **(b)** after IT injection and stained with Cresyl violet. Scale bar, 200 εm.

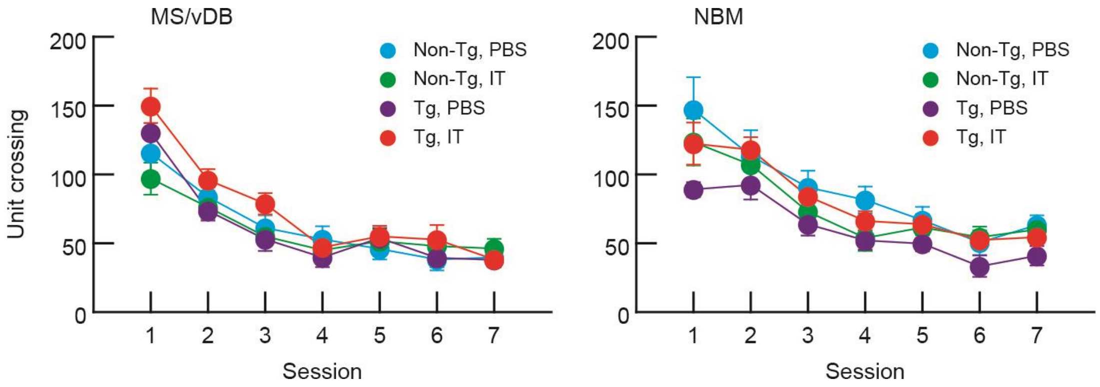

Supplementary Figure S3. Unit crossings during the exploration phase in the serial object exploration task. Unit crossing in the open field during each session was measured to validate spontaneous locomotor activity. It was gradually decreased along with the progress of sessions in a similar fashion among the four mouse groups that received the injection into the MS/vDB (group, $F_{3,28} = 1.199$, $P = 0.328$; session, $F_{6,168} = 71.393$, $P < 0.001$; interaction, $F_{18,168} = 1.883$, $P = 0.020$; two-way repeated-measures ANOVA, no significant simple main effect of group by *post hoc* analysis) or NBM (group, $F_{3,28} = 1.904$, $P = 0.152$; session, $F_{6,168} = 59.838$, $P < 0.001$; interaction, $F_{18,168} = 1.063$, $P = 0.394$, two-way repeated-measures ANOVA), indicating similar locomotor activity among the mouse groups injected either into the two basal forebrain regions.

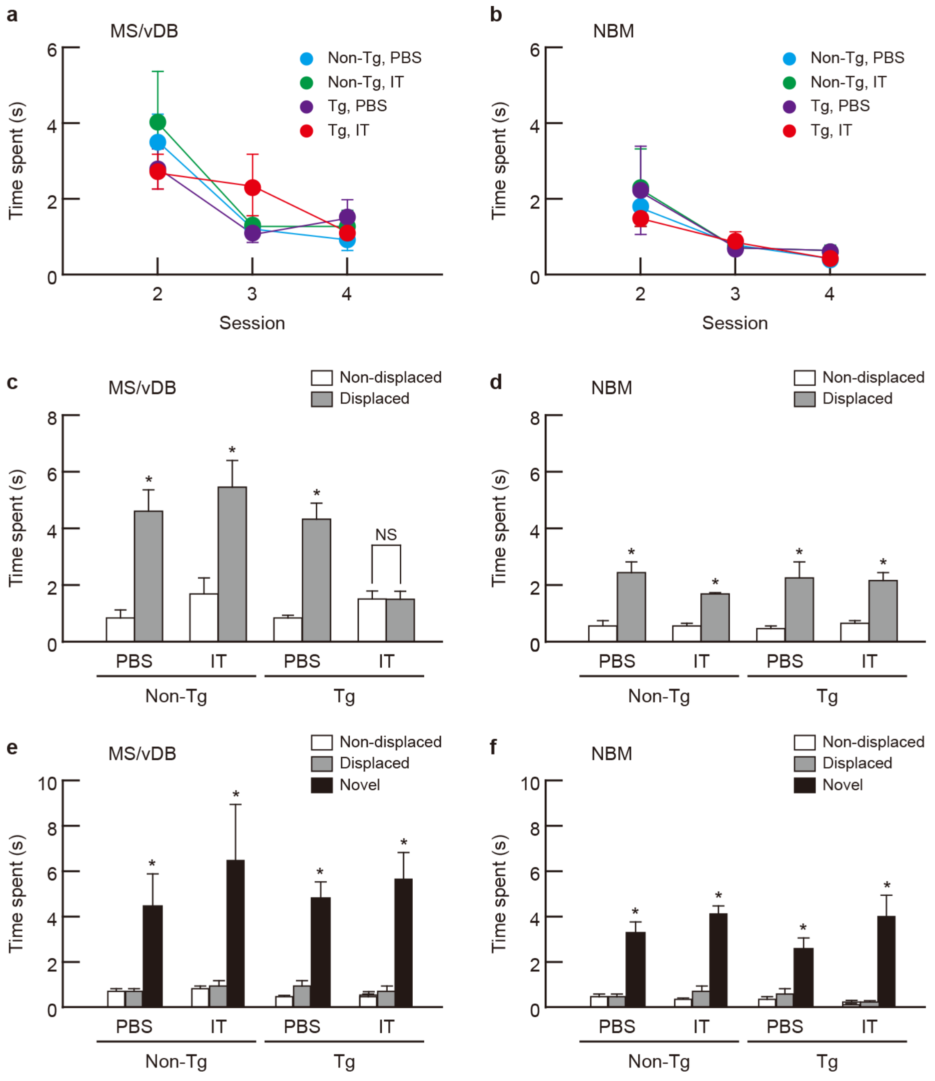

Supplementary Figure S4. Time spent in contacts with objects in the serial object exploration task. (a, b) Time spent in contacts with objects during S2ï S4. Tg and non-Tg mice were injected with IT solution or PBS into the MS/vDB (a) or NBM (b) and used for the task. Data are presented as mean ± s.e.m. *n* = 8 for each group. Throughout the sessions (S2ï S4), the time spent in contacts was similarly reduced among the four mouse groups with the injection into the MS/vDB (group, $F_{3,28}$ = 0.169, P = 0.916; session, $F_{2,56}$ = 19.612, P < 0.001; interaction, $F_{6,56}$ = 1.378, P = 0.240;

two-way repeated-measures ANOVA) or NBM (group, $F_{3,28} = 0.185$, $P = 0.906$; session, $F_{2,56} = 10.479$, $P < 0.001$; interaction, $F_{6,56} = 0.238$, $P = 0.962$; two-way repeated measures ANOVA). (c, d) Time spent in contacts with the non-displaced and displaced objects on a per-object basis in the spatial recognition test (S5). Mice injected with IT solution or PBS into the MS/vDB (c) or NBM (d) were used. Data are presented as mean ± s.e.m. $n = 8$ for each group. *$P < 0.05$ vs non-displaced object. NS, not significant. Two-way ANOVA for the MS/vDB injection indicated a significant main effect of group ($F_{3,28} = 4.263$, $P = 0.013$) and object ($F_{1,28} = 61.963$, $P < 0.001$) with a significant group × object interaction ($F_{3,28} = 6.836$, $P < 0.001$). The time spent with the displaced objects was significantly higher than with the non-displaced objects in the PBS- or IT-injected non-Tg and PBS-injected Tg mice ($P < 0.05$, Bonferroni method), whereas the value for the displaced and non-displaced objects did not differ in the IT-injected Tg mice. For the NBM injection, the time spent with the displaced objects was higher than with the non-displaced objects in each mouse group (group, $F_{3,28} = 0.847$, $P = 0.480$; object, $F_{1,28} = 86.828$, $P < 0.001$; interaction, $F_{3,28} = 1.110$, $P = 0.362$; two-way ANOVA). (e, f) Time spent in contacts with the non-displaced, displaced, and novel objects on a per-object basis in the object recognition test (S7). Mice injected with IT solution or PBS into the MS/vDB (e) or NBM (f) were used. Data are presented as

mean ± s.e.m. $n = 8$ for each group. *$P < 0.05$ vs either non-displaced or displaced object. Two-way ANOVA for the MS/vDB injection indicated a significant difference among objects (group, $F_{3,28} = 0.\ 328$, $P = 0.805$; objects, $F_{2,56} = 35.494$, $P < 0.001$, interaction, $F_{6,56} = 0.284$, $P = 0.942$), and the value for the novel object was higher compared to that for the familiar non-displaced or displaced object in each mouse group. For the NBM injection, the time spent was also significantly greater for the novel object than for the other two objects in each group (group, $F_{3,28} = 0.914$, $P = 0.447$; object, $F_{2,56} = 112.836$, $P < 0.001$; interaction, $F_{6,56} = 1.866$, $P = 0.103$; two-way ANOVA).
